# Image–text coherence and its implications for multimodal AI

**DOI:** 10.3389/frai.2023.1048874

**Published:** 2023-05-15

**Authors:** Malihe Alikhani, Baber Khalid, Matthew Stone

**Affiliations:** ^1^Department of Computer Science, University of Pittsburgh, Pittsburgh, PA, United States; ^2^Department of Computer Science, Rutgers University, Piscataway, NJ, United States

**Keywords:** coherence, discourse, multimodality, machine learning, evaluation

## Abstract

Human communication often combines imagery and text into integrated presentations, especially online. In this paper, we show how image–text coherence relations can be used to model the pragmatics of image–text presentations in AI systems. In contrast to alternative frameworks that characterize image–text presentations in terms of the priority, relevance, or overlap of information across modalities, coherence theory postulates that each unit of a discourse stands in specific pragmatic relations to other parts of the discourse, with each relation involving its own information goals and inferential connections. Text accompanying an image may, for example, characterize what's visible in the image, explain how the image was obtained, offer the author's appraisal of or reaction to the depicted situation, and so forth. The advantage of coherence theory is that it provides a simple, robust, and effective abstraction of communicative goals for practical applications. To argue this, we review case studies describing coherence in image–text data sets, predicting coherence from few-shot annotations, and coherence models of image–text tasks such as caption generation and caption evaluation.

## 1. Introduction

The internet has become a multimodal information ecosystem, where units of content—news articles, web pages, posts to social media—regularly tie together written words, emoji and other icons, static and dynamic imagery, and links to yet more multimodal content. Faced with the heterogeneity of online information, Artificial Intelligence (AI) researchers have increasingly characterized problems of information access from the perspective of multimodality: for example, producing text captions that make visual information more accessible (e.g., Lin et al., 2014; Young et al., 2014); or taking both text and image content into account in information retrieval (e.g., Funaki and Nakayama, 2015; Chowdhury et al., 2019). At the same time, this heterogeneity has empowered AI researchers to compile vast multimodal datasets (e.g., Sharma et al., 2018) and to build large scale “foundation” models (e.g., Lu et al., 2019; Radford et al., 2021) trained to capture cross-modal patterns and make cross-modal predictions.

Applications of such models, like the DALL-E system for synthesizing imagery from text (Ramesh et al., 2021), have captured the imagination of researchers and the public alike and serve as high-profile examples of the ability of representation learning to drive surprisingly rich AI capabilities.

**Figure 1:**
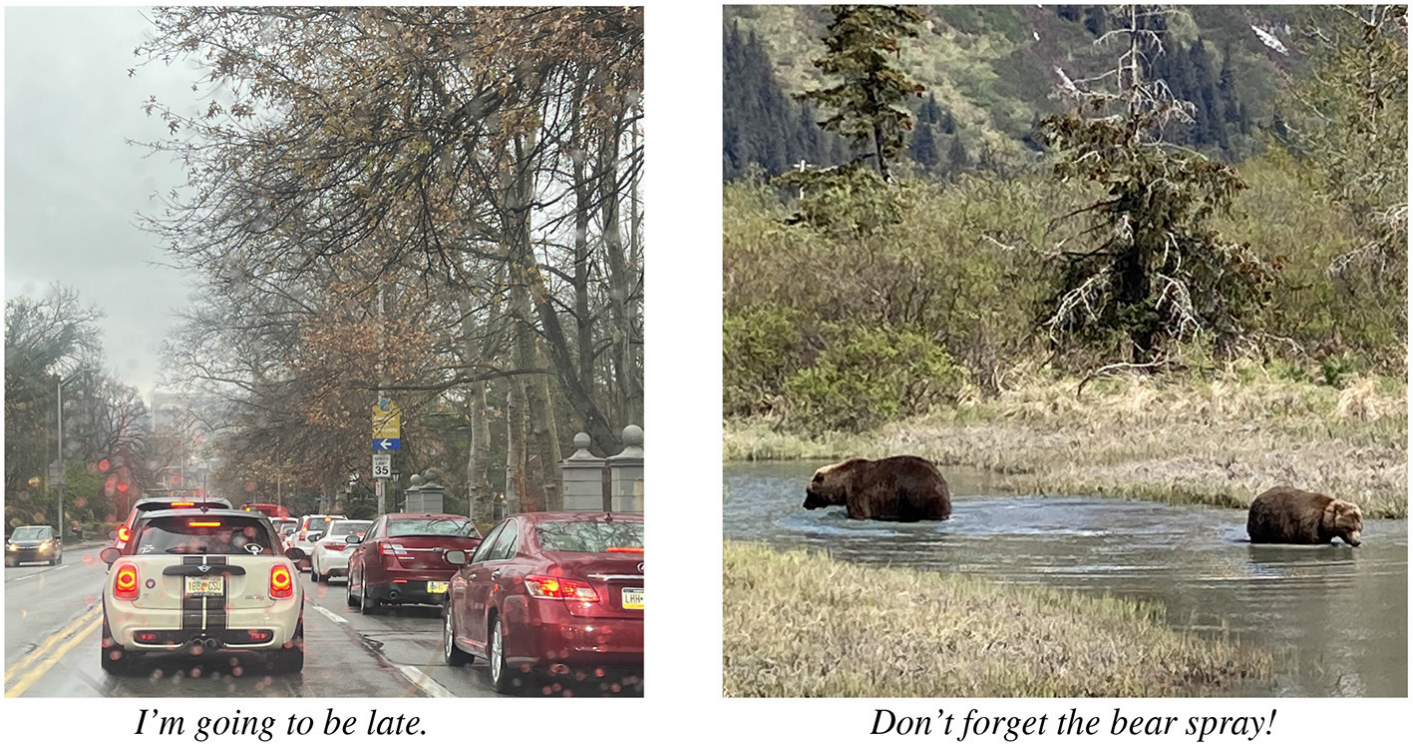
People combine text and images creatively to communicate. In these two unrelated image–text pairs, the images provide explanations for what is described in the text. Image credits: Malihe Alikhani.

The rapid progress in multimodal AI brings new urgency to the challenge of better understanding the data, tasks, model architectures, and performance metrics in the field. In fact, even the basic data points, “image–text pairs” scraped from the web, as in Radford et al. (2021), involve diverse and surprising juxtapositions. In Figure 1, for example, we see image–text pairs where the image depicts the *reason* for the text contribution—the depicted traffic is why the author will be late; the possibility of encountering bears in nature is why repellent is needed. Though such text is “grounded” in imagery only in a very abstract way, such inferences seem like common-sense to human readers. How good then are large vision–language models at capturing the varied implicit generalizations and relationships that connect text and imagery? How robust to this variation are machine learning approaches to using natural language as a supervision signal for multimodal inference? Can we design models that better understand and reason about these inferential links? More generally, what concepts and methods are needed for AI researchers to explore such questions in precise and effective ways?

In this paper, we address these challenges through the lens of theories of discourse coherence (Phillips, 1977; Hobbs, 1979, 1985; Asher and Lascarides, 2003). Our focus is on image–text coherence, where we argue that coherence relations that resolve the interpretation of text segments against juxtaposed imagery offer a broad and powerful framework to improve AI datasets, models, and systems so that they can better account for the structural, logical and purposeful organization of authors' communicative contributions to online discourse.

Coherence theory originates in the detailed analysis of the inferences needed to support text interpretation in knowledge-based approaches to natural language processing. For example, this discourse from Hobbs (1985 ex 3) depends on common-sense knowledge about books are formed and handled:

(1) John took a book from the shelf. He turned to the index.

Coherence here consists of the fact that the first event brings about the situation in which the second event takes place—a relationship referred to as *Occasion* (Hobbs, 1985) or *Narration* (Asher and Lascarides, 2003). For coherence theory, establishing this *Occasion* relationship guides and prompts key inferences, including the inference that John's turning involves opening the book he must be holding in his hand to a new page and the inference that the index refers to the section of this book John exhibits. While current AI rarely approaches such inferences explicitly, coherence theory nevertheless remains an influential paradigm that informs a wide range of AI work on text discourse, as we survey in Section 2.

Interpreting image–text presentations requires analogous inferences across modalities, including inferences that locate the viewpoint of imagery (Cumming et al., 2017), identify depicted objects (Abusch, 2013), and place the ongoing scene in the arc of the narrative (Cohn, 2013). Some coherence relations link imagery together (McCloud, 1993). Others guide inferences that enrich the joint interpretation of communicative actions across modalities (e.g., Lascarides and Stone, 2009a; Stone and Stojnic, 2015). The specific case of image–text coherence is the focus of our work here and underpins the contribution of our research. Section 2 builds on our review of broader work on coherence in AI to motivate and characterize image–text coherence.

Having laid out the principles of coherence, we go on to demonstrate the significance of image–text coherence for state-of-the-art multimodal AI. Section 3 explores how coherence can be used to annotate and to analyze image–text datasets. Section 4 illustrates how coherence can be used to make sense of the representations and learning of different model architectures for multimodal AI. Section 5 reviews how coherence-aware tasks and metrics enhance researchers' ability to build more useful tools and measure performance in more meaningful ways. We close by suggesting some key directions for building on these successes in future research.

## 2. Coherence in image–text presentations

We begin with an overview of coherence theory. We have two aims. Our first aim is to present the motivations, analyses, and principles of coherence theory as an approach to multimodal discourse. Like text discourse, we argue, multimodal discourse recruits numerous fine-grained inferences to enrich the interpretation of communicative contributions in context; what unifies these inferences is the need to establish coherence relations that organize the contributions of parts of the discourse into an integrated whole. It may seem counterintuitive that such an approach—a theory devised to address abstract foundational questions about meaning—should pave the way for concrete progress in AI. Our second aim, then, is to explain why the constructs of coherence theory have such direct, practical implications for AI methodology in general, and for multimodal AI in particular.

### 2.1. Coherence: The key ideas

Authors have different purposes in presenting information. The writer of Example (2), for example, uses the second sentence to offer an explanation of why the first event came about.

(2) Max spilt a bucket of water. He tripped on his shoelace.

Such relationships are central to making sense of the author's message. The second sentence of Example (2) works as an explanation, for example, only because we understand *He* as Max, *his* as Max's, the shoelace as that of one of the shoes Max must be wearing, and the event where *He tripped* as located temporally immediately prior to the spilling. Coherence theory counsels that we take a fundamentally relational view of actions in discourse. Successive actions in discourse don't express independent propositions or act unassisted to influence the audience: discourse contributions build on one another. Coherence relations specify how they do this. (Our discussion of Examples 2–4 follows Kehler, 2002; Asher and Lascarides, 2003). We counsel AI researchers also to take a relational approach to discourse actions. One lesson of our experiments in Section 4 is that AI architectures should not assume that discourse actions stand on their own. Instead, AI architectures should learn about discourse actions *via* their latent relation to other discourse actions in the context.

After all, relationships vary. Here are variants of Example (2) where the followup sentences make contributions of very different kinds.

(3) Max spilt a bucket of water. He spilt it all over the rug.
(4) Max spilt a bucket of water. John dropped a jar of cookies.

In Example (3), we find what Hobbs (1985) calls *Expansion*. We learn more about the initial event, its context and consequences. In Example (4), we find what Kehler (2002) calls *Resemblance*. The author presents another event as notable for its similarity and difference to the first. Coherence theorists have elegant ways to systematize the diversity of such relational interpretations in a rational taxonomy. For Kehler (2002), for example, Examples (2–4) illustrate *Cause-Effect, Contiguity*, and *Resemblance* relations (respectively), each a manifestation of the relationality and fitness of human thought. At the same time, AI research is increasingly successful at standardizing guidelines for annotators to classify the relations found in specific examples.

Strikingly, these different relations are not encoded directly in the linguistic forms and structures that make up Examples (2–4). Of course, the forms of referring expressions in 3 are the pronouns that you'd expect if this was a straightforward extended description of a single scene; the structure of Example (4) also exhibits the syntactic and semantic parallels you'd expect if the author wanted to facilitate a comparison. In that sense, linguistic form corroborates coherence. But form does not signal coherence—there are no words or constructions that give decisive evidence about what the author has in mind. Ambiguity is pervasive. A consonant lesson of Section 5 is that AI researchers should be skeptical that models and metrics learn and respect coherence. If coherence matters, rather than hoping learning methods capture it automatically, we recommend designers solve coherence-aware tasks and assess systems on coherence-aware metrics.

Alternative coherence relations provide broad organizing frameworks for interpretation, rather than clearly demarcated elements of form or content. Nevertheless, ambiguities in coherence often correlate transparently with clear interpretive differences. Smyth (1994) uses Example (5), to illustrate the connection between coherence and coreference.

(5) Phil tickled Stanley, and Liz poked him.

Coherence could organize (Example 5) in two ways. The second sentence could describe Liz's contingent reaction to the tickling. This suggests that Liz poked Phil, perhaps expressing disapproval of his action. Alternatively, the two sentences could describe similar events. That suggests that Stanley is the object of both tickling and poking. Asking how *him* is interpreted indirectly answers questions about coherence. Similarly, we can easily appreciate the different coherence relations in Examples (2–4) by considering when we understand the second event to have occurred: before the first, simultaneous to it, or at any time on the same relevant occasion. As we explore in Section 3, such interpretive effects enable AI researchers to approach coherence through a surprisingly diverse set of methodologies for data annotation and analysis.

Imagery, like language, must be understood by inference, and scholars have long argued that the interpretation of visual communication also aims to establish coherence. McCloud (1993), for example, argues that coherence relations between panels underpin storytelling in comics. Abusch (2013) makes an analogy between the persistence of individuals across panels in comics and the phenomenon of coreference in text discourse. Cumming et al. (2017) show how film viewers rely on coherence relations to infer the spatial relationships implicitly connecting the viewpoints of successive shots. In all cases, viewers must draw inferences about what objects imagery is depicting, where, and when—just as in they must draw inferences to understand linguistic content. In all cases, they use coherence to do it. Koby Leff's video essay presents a particularly clear visual explanation of the phenomenon; Alikhani and Stone (2018) analyze a case study of coherent visual communication in computational terms.

Text communication and visual communication thus share common principles of coherence, but we see the overarching role of coherence especially vividly when we consider the coherent relationships between text and imagery. Authors regularly juxtapose text and imagery to present their ideas. When we establish the coherence of such presentations, we can find that text relates to imagery; the text may describe how the imagery was obtained, what it shows, what its context is, or what its implications are. Conversely, we can use coherent links to text to establish what imagery depicts: perhaps a representative moment during the event described by the text, or perhaps a telling moment as the event got underway, or achieved its results. Such interpretations attest to the importance of the cross-modal coherence relations which we illustrated already with Figure 1 in the introduction. Coherence relations can provide a framework for ways that we pose questions about commonsense inferences in image–text presentations.

In fact, cross-modal coherence relations have a long history in the analysis of face-to-face communication (Engle, 2001; Lascarides and Stone, 2009a; Stone and Stojnic, 2015; Hunter et al., 2018; Hunter, 2019), as a formal tool to operationalize cognitive scientists' view that speakers use diverse modalities, including speech and coverbal gesture, to present integrated messages (McNeill, 1992; Bavelas and Chovil, 2000; Kendon, 2004). Image–text coherence is less well studied (the work of Feiner and McKeown, 1991, who used a taxonomy of cross-modal coherence relations to inform the automated synthesis of multimodal documents, is a prescient exception), but it provides new illustrations of the key principles of coherence.

**Figure 2:**
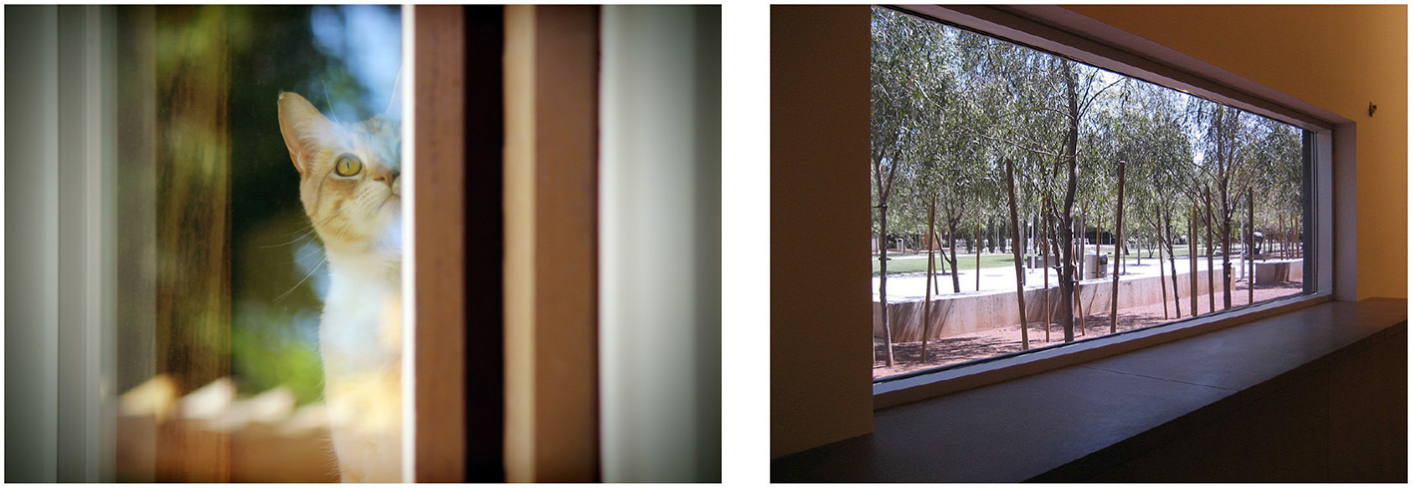
Two different ways of interpreting “looking out the window” as a specification of an image. **(Left)** Visual information describing the pose and activity of the subject in a photograph of a cat by Cristie Guevara (CC Public Domain 1.0 *via* publicdomainpictures.net). **(Right)** Meta-level information describing the camera viewpoint in a photograph of the Phoenix Art Museum by Chanel Wheeler (CC BY-SA 2.0 *via* Wikimedia Commons).

Consider the images of Figure 2 for example.

Both images are associated with the summary “looking out the window” but the text gets two qualitatively different interpretations. At left we have view of a cat through glass. It's the cat that's looking out the window. This is a characteristic example of a coherence relation we have called *Visible* (Alikhani et al., 2020); as we review in more detail in Section 3, we attribute many unique features of common image–caption corpora in AI research to the distinctive inferential character of text that supplies *Visible* information.

At right of Figure 2, meanwhile, we have a view of a window, framed to draw attention to the landscaped scene outside. The image features no gazing subject. It's the camera that's looking out the window. This is an example of a different coherence relation we have called *Meta* (Alikhani et al., 2020); utterances often get their coherence through *Meta-talk* by characterizing related communicative actions rather than by amplifying the communicated content of related segments (Hobbs, 1985; Sanders et al., 1992; Asher and Lascarides, 2003). *Meta* text doesn't summarize the visual information in the image like a typical caption would, but in some genres authors often supply *Meta* text to accompany their imagery. Such ambiguities in image–text coherence mirror the ambiguities found in text–text coherence. Text that accompanies imagery can provide qualitatively different kinds of information about the image it amplifies; there may be little surface-level information that reveals the contribution that text is making. Thus, there is a crucial but implicit role for coherence in interpreting image–text presentations, one that we argue impacts the data, models, tasks, and metrics of multimodal AI systems.

### 2.2. The methodology of coherence

Because of the ambiguity of coherence, annotated data is indispensable for AI experiments, whether in training models of coherence by supervised learning or in evaluating the predictions of unsupervised methods. Our work on text–image coherence is inspired by the success of analogous approaches to text discourse, particularly the theoretical work of Asher and Lascarides (2003) and the empirical work of Prasad et al. (2008).

To start, we need a framework that systematically organizes coherence relations based on their implications for the structure, content, and purpose of the discourse. Asher and Lascarides (2003) introduce such a taxonomy of coherence relations between discourse segments, as part of their Segmented Discourse Representation Theory, or SDRT. The simplest relations are based on reference to shared entities: Examples include *Expansion* (as in Example 3) when a second discourse unit amplifies and expands on what's described in the first unit, and *Narration* (as in one interpretation of Example 5), when a second discourse segment describes an event that follows the one described in the first segment.

SDRT also involves relations at proposition level, such as the *Parallel* relation that connects two discourse segments that express propositions that make similar claims about similar entities (as in one interpretation of Example 5).

Finally, SDRT includes relations that describe the intents and goals of the utterances—these are particularly important in interactive relationships such as *Correction* and *Clarification Request* that connect utterances by different speakers.

To annotate these relations, researchers have mapped out structured, multifaceted, hierarchical annotation guidelines (Prasad et al., 2008; Rohde et al., 2018; Alikhani et al., 2019). In general, specifying coherence relations first requires deciding how discourse elements attach into an ongoing discourse structure; see especially Webber et al. (2003). For each discourse unit, we need to describe what other units it's related to by coherent links. Connecting a discourse segment to a related segment can create a sibling (coordination) or a child (subordination), generating a hierarchical structure. Different discourse frameworks model the structure of the discourse differently. Some of them only capture shallow relations, while others, like SDRT, use more complex and hierarchical graphs.^1^ In extended multimodal presentations, like blog posts involving multiple images interleaved with extended textual descriptions (Alikhani and Stone, 2018) or contributions to spoken conversation including multiple utterances and co-verbal gestures performed in synchrony (Lascarides and Stone, 2009a), it's routine for each contribution to attach both to a synchronous contribution across modalities and a previous contribution in the same modality.

Our work on image–text coherence relations highlights cases whose discourse structure is relatively clear—for example, images together with their alt-text, an auxiliary record that is understood as providing subordinate, supplementary information to accompany the image. In more complex presentations, the question of multimodal discourse structure is a challenging issue ripe for further research.

Given an attachment, researchers first decide which of the top-level categories describes the relationship between the two segments (Prasad et al., 2008; Hunter et al., 2018). We have found it helpful to give annotators the option of specifying multiple top-level relations. Given a relation, we can then “drill down” to characterize the relationships in more detail. Webber et al. (2019) describes the hierarchical decisions that annotators must make to resolve the coherence relations defined for the Penn Discourse Treebank.

We can sometimes extend coherence annotation protocols to multimodal discourse by building on relationships that have been studied in text. The *Narration* relation (Hobbs, 1985; Asher and Lascarides, 2003) is a case in point. In multimodal discourse, two images are connected by the *Narration* relation when the second image shows the subsequent event of what's depicted in the first image. We can even find *Narration* from text to images and vice versa. An image can show what happened right after the text it elaborates, and text can report what happened right after the image it modifies. More generally, *Narration* is one of a family of coherence relations expressing contingent temporal connections that link an event to its *preparatory process, its culmination*, or *its result state*. These relationships can be found between *when*–clauses and main clauses in discourse (Moens and Steedman, 1988), between successive clauses in discourse (Webber, 1987), or between related text and imagery (Alikhani and Stone, 2018).

In other cases, we need new relations to describe inferences between text and images. In postulating such relations, we can draw valuable insights from research that explores how linguistic content can be related to other kinds of visual content. Engle (2001), for example, present a number of semantic relationships between speech and coverbal gesture, showing not only that gesture has a characteristic ability to relate to accompanying speech by *Depiction*, but that such relations combine with familiar relations from textual discourse such as *Exemplification*. Stone and Stojnic (2015), meanwhile, study how physical demonstrations are connected to speech and gesture, and appeal to relations such as *Summary*—where an utterance is understood to characterize a visible situation—and *Compliance*—where an event is understood to meet an expectation for action established by a previous utterance. As our empirical results in Section 3 make clear, presentations with different purposes and genres naturally feature distinctive relations, so we should expect future research to lead to broader perspectives on the range of possible coherence relations in multimodal communication.

**Figure 3:**
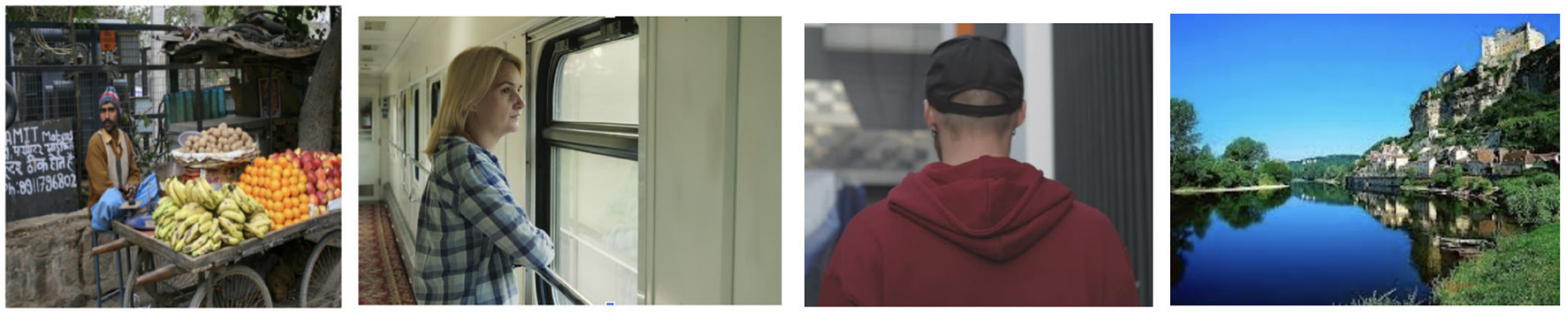
Captions from left to right: *A man sitting in front of a bunch of fruits. A woman is traveling on a train. The new manager of the team. The view from the bridge*. Text and images are linked together using a constrained set of coherence relations, which can summarize the structural, logical and purposeful relationships between the contributions of text and the contributions of images. Examples from the Conceptual Captions dataset (Sharma, 2020) that include Creative Commons Licensed image–text pairs.

Despite the many open questions about the structure and relations exhibited by coherent multimodal discourse, AI researchers can nevertheless make practical progress with coherence-based approaches. For text–image coherence, we have used a restricted list of relations to annotate data at scale (Figure 3), analyse large multimodal datasets, design coherence-aware models and evaluate our framework. Our taxonomy includes five relations—*Visible, Action, Subjective, Story* and *Meta*—any subset of which can define the coherent use of text to amplify on image content.

The *Visible* relation holds when the text presents information that is depicted in the image. This is similar to the *Restatement* relations in text (Prasad et al., 2008) in text, but here the content of an image overlaps with the content of its accompanying text. When the image depicts a moment or a snapshot of an action described in text, the pairs are connected with *Action* relation. The *Action* relation is analogous to *Elaboration* relations described in Prasad et al. (2008) for text.

The text and image are related with the *Subjective* relation when the overlapping information described in text sometimes includes an evaluative statement or a reaction to the content of the image. This is similar to *Evaluation* relations in text (Hobbs, 1985).

Similar to the *Occasion* relation of Hobbs (1985) that holds when a discourse unit provides the background for another discourse segment, sometimes the text provides a free-standing description of the occasion depicted in the image. We call this a *Story* coherence relation. Sometimes the text goes beyond just providing information about what's depicted in the image or the occasion. It describes how, when, or where the image was taken by explaining the presentation and production procedures. In such cases, we argue the text and the image are connected with a *Meta* coherence relation.

### 2.3. Why coherence?

The communicative functions of images and text in multimodal communication can be analyzed from several different perspectives. For example, Kruk et al. (2019) and Shuster et al. (2019) focus on the emotions evoked by presentations, Guo et al. (2019) study genre and style, and Otto et al. (2019) and Vempala and Preoţiuc-Pietro (2019) assess how text and images might be complementary or redundant.

The main contrast with our approach is that none of these frameworks try to model information-level inferences between text and images. An image might be a uniquely effective way to prompt emotion for example, but it would be surprising if our cognitive mechanisms could resolve ambiguity in the image (or in the accompanying text) to foster such affective engagement. Similarly, regardless of how we resolve their ambiguities, we will be able to classify related text and imagery as either complementary or redundant.

In text discourse, *information structure* is an alternative to coherence theory that provides yet another perspective to relate meaning to communicative goals. Information structure is a dimension of pragmatic meaning in language that helps explain variation in word order, intonation, and other linguistic cues that mark the relationship between utterances and their context (van Kuppevelt, 1995; Roberts, 2012). A key construct in theories of information structure is the “question under discussion,” or QUD; utterances relate to the context in part by addressing the QUD. Information structure describes how an utterance is partitioned into material that evokes the question under discussion and the material that supplies the answer. Although information structure is signaled grammatically, the point of emphasis of the speaker often has to be inferred (Bolinger, 1972). This makes information structure and coherence complementary: in particular, Hunter and Abrusán (2017) argue that coherence provides a flexible and perspicuous way of mapping the inferences involved in disambiguating information structure and reconstructing the QUD. Coherence is especially natural when we consdier how approaches should generalize to multimodal discourse, because there isn't anything analogous to information structure in a photograph, map, or diagram. Even the emphasis you find in gesture is very different structurally and compositionally from information structure in language (Kendon, 2004). Neither QUD nor the frameworks that prioritize ways that images serve complementary roles for text would give insights into inferences that connect the content of text and imagery. They do not provide a framework that can support data creation and model designs that we describe in the next section.

## 3. Coherence as a framework for analyzing image–text datasets

Our first argument for the utility of the coherence framework comes in characterizing AI datasets. Coherence relations can provide information about the inferential and linguistic characteristics of image–text corpora. In particular, coherence sheds light on how genres vary and how linguistic form is likely to change across datasets. In addition to characterizing the challenges of image–text inference, such results can also inform AI research by helping to define the domain adaptation that will be necessary to generalize machine learning results from one image–text collection to otheres.

In this section, we first describe the Clue dataset, which is the largest image-text dataset annotated with coherence relations. Then we discuss empirical studies that support the importance of coherence relations and their associated linguistic constructs in supporting commonsense inference. Finally, we discuss results on the correlations of coherence relations and genre, and the use of coherence relations to diagnose mismatches between image–text corpora and machine learning models.

### 3.1. Clue: A dataset of image–text coherence relations

Alikhani et al. (2020) presented Clue that provides a protocol for annotating coherence relations in text and imagery. They used the protocol to annotate from the 10,000 image-text pairs from the Conceptual Captions (Sharma et al., 2018) and Open Images (Kuznetsova et al., 2020) datasets. Half of these pairs include captions generated by models and half of them are captions that users have written for the images. Guidelines are described in details in Alikhani et al. (2020).

**Figure 4:**
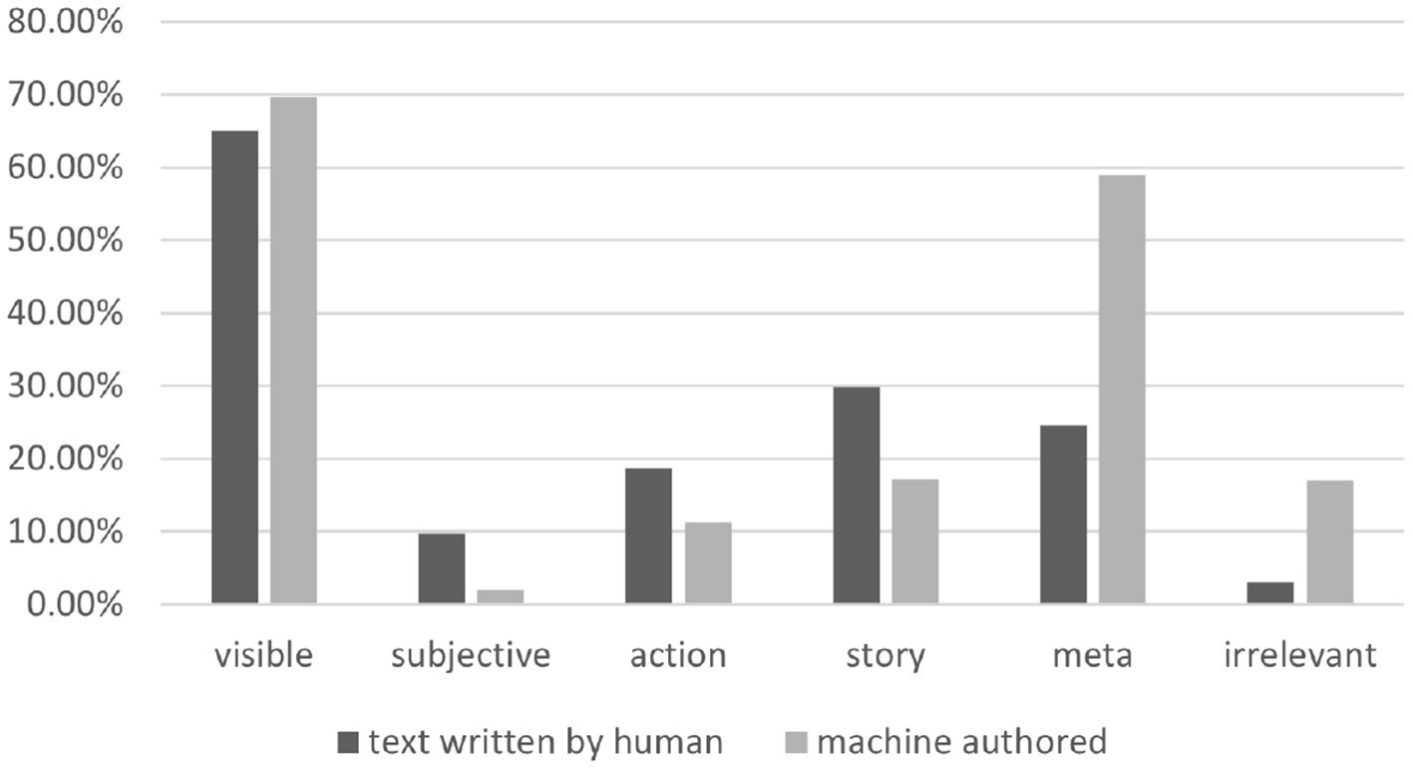
The distribution of coherence relations in our dataset.

Figure 4 shows the statistics over the resulting annotations presented in Alikhani et al. (2020). We can see that models struggle to generate subjective or story-like captions. The rate of captions and images with the *Meta* relation however is higher in text generated by models that signals the potential context hallucination problem.

### 3.2. Coherence predicts linguistic form

Alikhani and Stone (2019) present an empirical investigation and argue that we can learn new perspectives on commonsense inference by correlating coherence relations with linguistic constructs. In particular, they report that visible descriptions are very distinctive. They only describe what's depicted in the image in a restricted way.

They observed that the rate of captions that describe ongoing events (**atelic** events) is drastically higher than the rate of captions that describe events with end points (**telic** events). This is the difference between *arriving at an event* (telic) and *standing somewhere* (atelic). **stative** descriptions are also very common in captions. Many captions describe quality, condition or the state of what's depicted in the image. Examples include *the kids are happy* or *green bananas are on the table*. Alikhani and Stone (2019) argue that these captions are connected to images by the **illustration** or **visible** relation. They study the following datasets with different types of textual descriptions with images: (1) Google's Conceptual Captions (CC) (Sharma et al., 2018) (2) Flickr30K (Flickr) (Young et al., 2014) (3) Visual Storytelling (VIST) (Huang et al., 2016) (4) the Recipe dataset (Yagcioglu et al., 2018). While Flickr and COCO are captaining corpora, VIST includes story-like descriptions for connecting five images and CC pairs web images with relevant text from associated alt-text HTML attributes.

They studied 1,000 random image-text pairs from each of the discussed datasets. Over 94% of the text in caption corpora such as Flicker and COCO include atelic events. However, the rate drops to 40% in other image-text corpora, such as the multimodal recipe dataset. Alikhani and Stone (2019) includes the experiments and statistics details.

### 3.3. Coherence relations indicate Genre

AI models do not, by default, exhibit the behavior of the texts that they are trained on. As we can see in Figure 4, machine-generated text includes more captions with the meta relation in comparison with text written by humans. This is one of the main sources of the hallucination problem when the model includes information in generated text that may not necessarily be true. Information about the background or context of the image that is not depicted in the image. The coherence framework can help us identify, characterize and address these issues.

**Figure 5:**
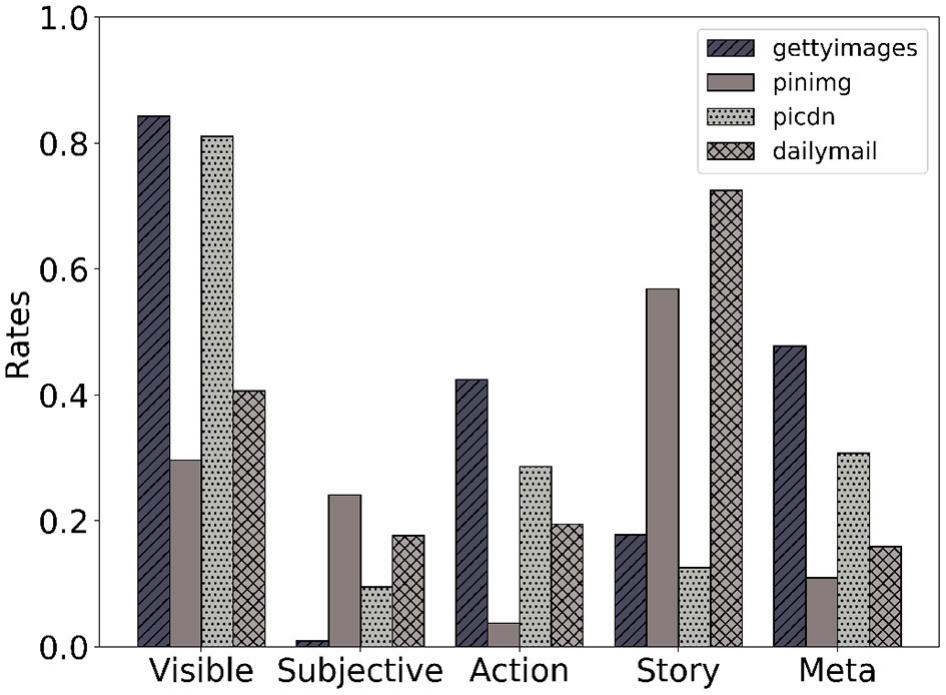
Different websites have different types of image–caption pairs (Alikhani et al., 2020).

Coherence relations indicate discourse goals. Figure 5 shows that the labels that our dataset presents correlate with the genre under which the captions have been produced, which means that text and images from different types of publications have different distributions of relations. For example, captions from a news website such as daily mail are more story-like, whereas Getty Images posts often include *Visible* captions.^2^

## 4. Using coherence to critique image–text models

In the previous Section, we discussed how coherence defines what linguistic forms and inferences go into text–image interpretation. We also discussed how the framework could identify weaknesses in machine learning models trained on image-text corpora. In this Section, we study how different model architectures and training mechanisms can capture coherence to various degrees. We first present the details of our computational experiments then move on to describe the results and discussions. Our computational investigations reveal that large multimodal models cannot accurately represent coherence relations. They fail to reason flexibly about the links that connect text–image pairs in the same way we described in our data.

### 4.1. Experiment structure

In this section, we describe our experimental setup, the problem formulation and then go on to describe the insights we draw from our results.

We investigate whether the two most recent and successful versions of these models, VilBERT (Lu et al., 2019) and CLIP (Radford et al., 2021) can implicitly learn coherence relations in image–text presentations during the pre-training process.

**VilBERT** is a transfomer based model pre-trained on the Conceptual Captions dataset. It is inspired from the BERT architecture and takes as input a sequence of image blocks and text tokens belonging to the image caption. Image blocks and text sequence are separated by a special token. It is trained using two proxy tasks: masked multi-modal modeling and multi-modal alignment prediction and then the learned weights. It relies on the self-attention mechanism of transformer architecture to learn the relations between different parts of an image and the corresponding textual tokens.

**CLIP** is trained using contrastive learning to maximize similarity text and image pairs. Unlike VilBERT, it has two separate encoders for the text and visual data. Visual encoder is either a variation of ResNet architecture or a visual transformer while the text encoder is based on the transformer architecture. A dot product between the text and image representations is used to compute the similarity and then loss is computed using the binary cross entropy objective.

#### 4.1.1. Problem formulation

The problem is structured in the form of a classification task where goal of the model is to classify the representation *h*_*i*_ as a coherence relation. We measure the model performance using the *F1* metric as it is a well-known metric for measuring a classification model performance.

Formally, speaking, we define a function *f*_θ_*p*__ as a pre-trained model which maps an input pair (*x*_*i*_, *v*_*i*_) to a vector *h*_*i*_ in a *d* dimensional representation space *R*^*d*^, where θ_*p*_ represents the pre-trained weights, *x*_*i*_ and *v*_*i*_ represent the text sample and its corresponding image respectively. To check whether the representation *h*_*i*_ has a certain information, we can define a linear probe *f*_*lin*_ which takes *h*_*i*_ as input and outputs a probability distribution *P*(*l*_*i*_|*h*_*i*_) over a given set of *n* labels *l*_*i*_ ∈ *L*. By tuning the weights associated with the linear probe *W*, we can learn whether the representation *h*_*i*_ to identify different classes *l*_*i*_ ∈ *L*.


(1) $$\begin{array}{l} \begin{array}{c} P\left(l_{i}|h_{i}\right)=f_{lin}\left(f_{\theta_{p}}\left(x_{i},v_{i}\right)\right)\\ f_{lin}\left(h_{i}\right)=\sigma \left(Wh_{i}+b\right)\\ L_{i}=\frac{1}{n}\underset{l_{j}\in L}{\sum }-log\left(P\left(l_{j}|h_{i}\right)\right) \end{array} \end{array}$$

Where $L_{i}$ is the cross entropy loss signal associated with the image-text pairs and their corresponding labels, and σ represents the sigmoid function. Since the parameters θ_*p*_ associated with the pre-trained model are frozen, the linear probe can only make use of information encoded in the representation *h*_*i*_.

To discern if the representations *h*_*i*_ encode the discourse relation information, we compare probes for the above mentioned models with the probes trained using representations from ResNet and BERT. In addition, we also fine-tune the pre-trained model weights θ to observe the improvements when the representations *h*_*i*_ are also fine-tuned. Our hypothesis is that pre-trained representations containing signals for identifying discourse relations should outperform the baselines and show competitive performance when compared to the models obtained using fine-tuned representations.

#### 4.1.2. Experiment parameters

As described in the Section 4.2, we fine-tune the models in two ways. When the pre-trained weights are frozen and only the probe weights are trained, we rely on a batch size of 64 and a learning rate of 5*10^−3^. However, when the we run the experiments to fine-tune the pre-trained weights we rely on a batch size of 8 and a learning rate of 10^−5^. In both cases, the weights are optimized using the Adam optimizer and a small regularization weight of 10^−5^ is utilized to prevent model from overfitting the data.

### 4.2. Results

To conduct our experiments we split the 5760 image-text pairs into train, validation and test ratios of 0.85, 0.075, 0.075 respectively. The idea of our evaluation setup is to investigate whether the representations learned by the pre-trained models encode the discourse relations between image-text pairs. We do so by comparing the performance of fine-tuned models with their respective linear probes. If the fine-tuned models perform better than their respective linear probes it shows that pre-trained representations lack key information which would have allowed them to identify coherence relations and vice versa.

As stated earlier we build probes for two pre-trained models—CLIP and Vilbert. For the pre-trained Vilbert weights, we build two probes: for the visual-linguistic representations and linguistic representations outputted by the model. Visual-linguistic representations are obtained by combining the visual and linguistic representations, and linguistic representations use co-attention mechanism with image representations to represent relations between image and text pairs. For the CLIP model, we concatenate the visual and text representations to obtain the representation for image-text pair.

We present the linear probe performance based on the F1 average scores (macro and micro) and their breakdown in the Table 1. These results highlight the capability of representations learned by different architectures in encoding information necessary to identify discourse relations. Our results show that the probe for linguistic representations learned by the VilBERT using co-attention with image representations (VilBERTLinguistic) shows better performance when compared to the probes for other architecture including visual-linguistic variation of VilBERT. This could be a signal that architectures which try to learn fine-grained relations between image-text pairs are more suited to learning discourse relations.

**Table 1:** This table shows the F1 score averages and their breakdowns obtained by the linear probes for different visual-linguistic architectures.

**Model**	**Metrics**					
	**Macro average**			**Micro average**		
	**F1**	**Precision**	**Recall**	**F1**	**Precision**	**Recall**
CLIP	0.466	0.468	0.571	0.762	0.744	0.781
VilBERTVil	0.582	0.545	0.712	0.780	0.758	0.804
VilBERTLinguistic	**0.635**	0.601	0.713	**0.808**	0.796	0.820
Resnet	0.333	0.337	0.345	0.682	0.617	0.763
BERT	0.623	0.590	0.776	**0.798**	0.791	0.804
BERT + Resnet	**0.637**	0.614	0.712	0.794	0.798	0.789

Macro average gives equal weights to the model performance in each class and hence represents the effect of imbalance while micro average represents the overall average performance. The difference in performance highlights that representations learned by some model architecture are better suited for the purpose of identifying discourse relations; VilBERTLinguistic representations in this case.

When compared with the baseline probes, specifically BERT probe, the performance of the best pre-trained visual-linguisitic probes is similar in terms of the F1 score achieved as shown in the Table 1. This highlights that pre-trained models are unable to make use of the visual information in a meaningful manner to successfully encode the relations between the image-text pairs in the higher dimensional embedding space.

**Figure 6:**
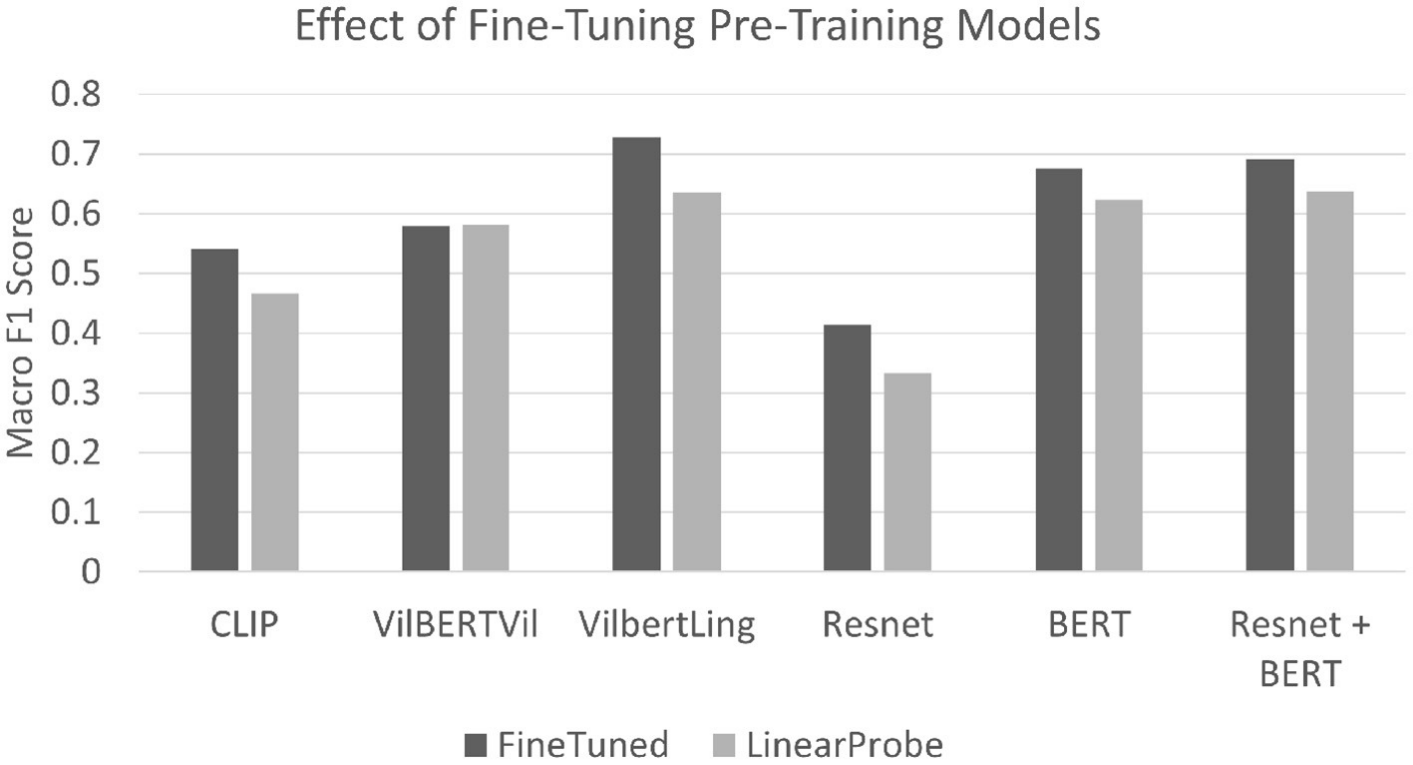
This figure highlights the change in macro F1 scores when the model weights are fine-tuned during the training process. In all cases, the performance for the fine-tuned models improves by a significant margin reflecting that pre-trained representations do not encode information necessary to identify discourse relations.

When the pre-trained model weights are fine-tuned we see a significant increase in the macro F1 score as shown in the Figure 6. Even though the best performance after fine-tuning is achieved by the VilBERTLinguistic model, it only lags behind the BERT + Resnet performance by a slight margin of 3%. This provides evidence that pre-training with large corpora of image-text pairs does not implicitly allow models like VilBERT and CLIP to encode discourse relations in the higher dimensional embedding space and calls for the exploration of techniques which explicitly utilize discourse relations to learn models whose predictions are in-line with human judgements.

## 5. Using coherence to define and evaluate AI systems

In this section, we discuss ways to use the dataset and models we discussed in the previous sections in designing multimodal systems. Previous works have shown the utility of using the coherence framework in design image retrieval models (Alikhani et al., 2022), caption generation models (Alikhani et al., 2020), automatic evaluation (Inan et al., 2021), and diagram understanding (Hiippala et al., 2021).

Can a coherence-aware model present information that is aligned with the goal of the discourse? Can a coherence-aware model significantly improve caption quality? Can we design automatic learned generation metrics that can evaluate the output of coherence-aware generation models?

### 5.1. Generating coherent captions

Can we design controllable image description generation models that can generate captions with respect to different coherence relations? Alikhani et al. (2020) introduced such controllable model using the Clue dataset. They used Transformer Networks (Vaswani et al., 2017) and designed a generation model that can output captions using a sequence-generation approach. The result is a sequence of sub-tokens that create the desired caption. The input includes different images features and the target coherence relation label. The relation label is the start token of the Transformer decoder.

The proposed model is able to reduce noise by around 30% from the generated captions overall. It includes substantially fewer irrelevant captions, and it can respect the discourse goals by generating captions connected to the image by the desired coherence relations. The success rates of the model when it was asked to generate visible, meta, story and subjective captions ware respectively 79.85%, 46.49%, 58.8% and 45.00%. The details can be found in Alikhani et al. (2020).

### 5.2. Coherence-aware learned evaluation metrics

As we observed in the previous section, image captioning metrics have struggled to give accurate learned estimates of the semantic and pragmatic success of output text. Inan et al. (2021) proposed the first discourse-aware learned metric for evaluating such properties in image descriptions. The goal of the metric is to output a score that reflects the quality of the generated caption given the image, coherence relation and the reference caption. In what follows we review the proposed model and results.

They worked with 1,000 image text pairs from the Conceptual Captions (CC) training dataset (Ng et al., 2020) and collected ratings for them. They use the cc imges as inputs to caption generation model presented by Alikhani et al. (2020). The model generates coherence-aware descriptions for these images in different coherence classes. Then, they asked the annotators to write *Visible* captions for 1,000 images from the OpenImages dataset (Kuznetsova et al., 2020) and called the dataset COIN (**C**orpus of **O**pen**I**mages with **N**atural descriptions).

The proposed approach has two versions—a baseline Vanilla version and a ViLBERT-based model. Both are trained on RaCCoontraining data with normalized human annotated rating to obtain the model's target score. Details of the dataset and ablation studies are available here (Inan et al., 2021). Table 2 presents the results of the COIN-based study. The last row shows the Kendall correlation coefficient between the scores assigned by users and the metric. The N-gram based metrics cannot adapt to the out-of-domain ground-truth captions from COIN which results in low correlation coefficients. The CIDEr scores have negative correlation coefficients which indicate negative association with user ratings. BLEURT and BERTScore do a much better job in comparison with CIDEr and N-gram based metrics but they are still agnostic to the coherence relation label. Our proposed model which is coherence-aware has the highest correlation scores with user judgments.

**Table 2:** Scores for different image captioning models as evaluated by users and different captioning metrics (Inan et al., 2021).

**System**			**Metrics**											
**Model**	**Coh. Label**	**Avg. Hum. Rating**	**B**_1_	**B**_2_	**M**	**R**_*L*_	**C**	**S**	**BR**	**BS-F**	**COSMic Vanilla**	**COSMic VBERT**	**COSMic Vanilla+**	**COSMic VBERT+**
BUTD	Visible	2.191	0.163	0.077	0.049	0.160	0.092	0.030	–0.877	0.863	0.706	0.796	0.522	0.641
Base	Visible	30.532	0.050	0.025	0.019	0.066	0.020	0.002	–1.114	0.862	0.696	0.777	0.516	0.614
	Meta	3.213	0.041	0.000	0.012	0.063	0.012	0.000	–1.059	0.863	0.548	0.727	0.505	0.602
	Subj.	2.830	0.033	0.012	0.011	0.057	0.017	0.000	–1.197	0.849	0.323	0.421	0.358	0.403
	Story	2.915	0.029	0.000	0.017	0.058	0.013	0.000	–1.304	0.842	0.533	0.629	0.482	0.527
Lite	Visible	3.298	0.028	0.011	0.013	0.053	0.011	0.000	–1.101	0.863	0.684	0.784	0.515	0.604
	Meta	2.830	0.026	0.010	0.008	0.055	0.015	0.000	–1.084	0.859	0.548	0.748	0.511	0.565
	Subj.	2.298	0.039	0.012	0.019	0.066	0.024	0.003	–1.217	0.849	0.364	0.451	0.379	0.419
	Story	2.426	0.036	0.000	0.018	0.062	0.021	0.000	–1.362	0.842	0.568	0.666	0.499	0.519
**Kendall's Correlation (τ)**		1.000	0.071	0.154	0.036	–0.036	–0.571	–0.052	0.286	0.445	0.571	0.546	0.667	**0.764**

The COSMic ViLBERT+ model has the highest correlation scores with human judgments.

## 6. Conclusion

When authors combine text and imagery, they use the different modalities of communication in concert: common principles of coherence relate communicative actions together, guide interpretive inferences, and resolve ambiguities. In this paper, we have described how the well-known theory of coherence in text discourse extends to image–text presentations and can guide AI research on mulitmodal communication. While we have thus far offered a range of findings to show the potential benefits of coherence in multimodal AI, we are optimistic for further research progress in all of these areas.

In particular, we have seen that coherence relations offer important tools to analyze data sets; coherence relations can allow us to quantify differences in language use across different corpora and even to explain the distribution of linguistic phenomena in corpora as a function of the distinctive character of coherence in corpora. Work on taxonomies of coherence relations for multimodal discourse is in its infancy. New genres and tasks could highlight the importance of additional relations or further distinctions in how text relates to imagery. Conversely, we have said little about imagery that gets its coherence from accompanying text. Alikhani et al. (2019) annotates the inferences that ground imagery in a specific domain through particular temporal, spatial, and logical connections, rather than through a traditional taxonomy of coherence relations. It is an open question whether coherence can be systematized more generally across images and text. Another challenge is accounting for the structure of multimodal discourse, particularly for presentations that involve relations within and across modalities. Multimodal analyses of situated conversations have revealed many complexities (Lascarides and Stone, 2009b; Hunter et al., 2018).

We have also seen that coherence relations also offer a valuable lens to critique and improve the architecture of machine learning models. Models that build in an assumption that text and imagery relate in simple, uniform ways, are less effective in capturing coherence than models that allow for more flexibility. Research in large vision–language models, however, has overwhelmingly designed around capturing the relationships between image content and *Visible* text. Relaxing this assumption offers exciting prospects for learning more powerful representations of the meanings of image–text presentations.

Finally, we have offered an example of coherence-aware tasks and evaluation metrics. Since current AI technology struggles with coherence, we need to take coherence into account from the start as we design and test AI systems. AI researchers still face many challenges in extending information and interaction tasks from their origins in text processing to multimodal communication. All of these domains, we believe, offer fruitful settings to pursue coherence-aware methodologies.

## Data availability statement

The original contributions presented in the study are included in the article/supplementary material, further inquiries can be directed to the corresponding author.

## Ethics statement

The studies involving human participants were reviewed and approved by Institutional Review Board, Rutgers University. The patients/participants provided their written informed consent to participate in this study. Written informed consent was obtained from the individual(s), and minor(s)' legal guardian/next of kin, for the publication of any potentially identifiable images or data included in this article.

## Author contributions

MA led data annotation, empirical analysis, and evaluation. BK led model training and implementation. MS led theoretical analysis, model design, and explanation. All authors contributed to the article and approved the submitted version.

## Footnotes

^1^Researchers in text discourse often focus on tree discourse structures (Hobbs, 1985; Kehler, 2002) but the analysis of multimodal discourse benefits from appealing to more flexible structures (Hunter et al., 2018).

^2^Getty Images: http://www.overleaf.com. Daily Mail: https://www.dailymail.co.uk/ushome/index.html.
